# Study of the Mechanical Recycling on the Properties of Glass Fiber-Reinforced Aliphatic Polyketone Composites

**DOI:** 10.3390/polym17202743

**Published:** 2025-10-14

**Authors:** Annamária Polyákné Kovács, Yitbarek Firew Minale, Mariann Éva Hegedűs, Tamás József Szabó

**Affiliations:** 1 Institute of Energy, Ceramics and Polymer Technology, University of Miskolc, Egyetemvaros, 3515 Miskolc, Hungaryhegedusmanka@gmail.com (M.É.H.);; 2 Department of Chemical Engineering, Bahir Dar Institute of Technology, Bahir Dar University, Bahir Dar 6000, Ethiopia

**Keywords:** recycling, mechanical recycling, degradation, engineering plastic, glass fiber composite, injection molding

## Abstract

This study aims to evaluate the effects of repeated mechanical recycling on the properties of a novel aliphatic polyketone composite reinforced with 15 wt% and 30 wt% glass fibers (PK15GF and PK30GF), providing insights into its potential for sustainable engineering applications. The investigation focuses on three main aspects: changes in melt flow index (MFI) and viscosity, the influence of glass fiber content on thermal and mechanical stability, and the retention of structural integrity and crystallinity under multiple processing cycles. Composites, commercially available since 2019, were subjected to single- and five-cycle recycling with 100% reprocessed content. Comprehensive characterization—including tensile testing, Differential Scanning Calorimetry (DSC), Dynamic Mechanical Analysis (DMA), Fourier Transform Infrared Spectroscopy (FT-IR), Melt-Flow Index (MFI), Differential Thermal Analysis (DTA), and mechanical tensile testing—revealed filler-dependent alterations in morphology, thermal stability, and crystallinity. MFI decreased from 100.56 to 42.63 g/10 min for PK15GF, indicating pronounced chain scission, recombination, and crosslinking, whereas PK30GF decreased only from 89.00 to 59.76 g/10 min. FT-IR spectra confirmed greater crosslinking in PK15GF, while DSC and DMA demonstrated smaller T_g_ and ΔHm variations in PK30GF (T_g_ +0.45 °C, ΔHm −13.93 J·g^−1^) versus PK15GF (T_g_ +1.13 °C, ΔHm −69.24 J·g^−1^). These findings reveal that higher glass fiber content mitigates degradation, preserves structural integrity, and maintains thermal and viscoelastic stability, establishing clear correlations between filler content, mechanical performance, and recyclability. Overall, this work provides mechanistic insights into degradation pathways and demonstrates the potential of glass fiber-reinforced aliphatic polyketones for sustainable, high-performance engineering and automotive applications.

## 1. Introduction

The growing global concern regarding plastic waste and resource depletion has placed increasing pressure on industries and policymakers to implement sustainable solutions across the entire plastics value chain [1]. Among these, mechanical recycling has emerged as one of the most efficient and scalable strategies for managing post-consumer and post-industrial polymer waste, enabling materials to re-enter the production cycle with minimal environmental burden [2]. While early recycling efforts largely focused on commodity polymers such as polyethylene (PE), polypropylene (PP), and polyethylene terephthalate (PET), recent trends demonstrate a clear shift toward extending circular economy strategies to high-performance engineering polymers [3,4].

The demand for engineering plastics with superior mechanical, thermal, and chemical properties in the automotive, aerospace, and electronics industries has accelerated the development of novel polymer systems with enhanced functionality [5]. At the same time, the need for sustainable solutions has created strong incentives to evaluate the recyclability of these advanced materials [6]. Recent reviews emphasize that while mechanical recycling of engineering polymers is feasible, degradation processes such as chain scission, oxidation, and morphological instabilities remain critical challenges that limit performance retention [2].

Among the emerging engineering thermoplastics, aliphatic polyketones (PKs) have received increasing attention due to their highly regular molecular structure, high crystallinity, toughness, and outstanding chemical resistance [7,8]. A notable milestone occurred in 2019, when a novel linear ethylene–propylene-based polyketone was synthesized using a palladium-based catalyst system and scaled up for industrial production by Hyosung Chemical Ltd. in Ulsan, South Korea [9,10]. This new class of polyketones exhibits a unique balance of mechanical strength, dimensional stability, and chemical resistance, making it a promising candidate for applications in the automotive and engineering sectors, where sustainability is increasingly prioritized [11].

However, the successful integration of polyketones into a circular economy model requires a thorough understanding of their degradation behavior and property evolution under repeated recycling cycles. Reinforcement with short glass fibers is a widely adopted strategy to enhance stiffness, dimensional stability, and load-bearing capacity, but it also introduces complexity into melt processability, fiber–matrix adhesion, and recyclability [12,13]. For instance, studies on polypropylene/glass fiber composites have shown that repeated recycling leads to pronounced reductions in melt-flow index (MFI), fiber breakage, and deterioration of interfacial adhesion.

More broadly, recent work on polymer composites highlights that while fiber reinforcement improves mechanical performance, it also accelerates morphological changes and modifies viscoelastic behavior during reprocessing [14,15].

In high-performance thermoplastic composites, such as carbon fiber-reinforced systems, multiple recycling cycles have been shown to induce fiber shortening, matrix degradation, and property loss, further illustrating the complexity of recycling fiber-filled engineering materials.

Despite these advances, data on the recyclability of glass fiber-reinforced aliphatic polyketones remain scarce. The purpose of this study is to systematically investigate the effects of multiple mechanical recycling cycles on the thermal, viscoelastic, and mechanical behavior of PK/GF composites. This work falls within the broader field of sustainable recycling of high-performance thermoplastic composites and addresses the critical need to understand degradation pathways, property retention, and the potential applicability of these materials in structural and automotive applications. To achieve this, composites reinforced with 15 wt% and 30 wt% glass fibers were subjected to one and five recycling cycles under identical injection molding conditions, using 100% reprocessed content.

## 2. Materials

The material analyzed in this study is a glass fiber-reinforced aliphatic polyketone (PK) composite, containing either 15 wt% or 30 wt% short glass fibers (average fiber length: 400–600 μm). The composites have densities of 1.35 g/cm^3^ (15 wt% GF) and 1.45 g/cm^3^ (30 wt% GF), respectively [9,16,17]. These materials are designed to improve mechanical performance and are suitable for injection molding applications [18]. The PK matrix is based on an ethylene–propylene copolymer (EPCO) synthesized via palladium-catalyzed copolymerization of carbon monoxide, ethylene, and propylene monomers [9]. This particular grade, designated as M630F, was developed by Hyosung Chemical Corporation. The novel catalyst system was introduced in 2013, and the industrial production facility was completed in Ulsan, South Korea in 2015 [19,20]. The first commercial-scale production began in 2019 [15]. The resulting polymer exhibits a linear and perfectly alternating structure composed of carbon monoxide and α-olefin units, typically represented as (–CH_2_–CH_2_–CO–)_n_ and (–CH_2_–CH(CH_3_)–CO–)_m_ [21,22]. According to the manufacturer’s technical data sheet (TDS), the melt temperature of the material is approximately 240 °C, and its glass transition temperature (T_g_) is around 15 °C [23].

### 2.1. Preparation of the References and Recycled PK-GF15 and PK-GF30 Composite Samples

Recycled aliphatic polyketone composites were prepared by mechanical recycling processes consisting of regranulation followed by reinjection molding steps in three different recycling cycles of 15% and 30% glass fiber content. The sample codes and formulations are given in Table 1. The following process steps were applied in order to obtain these composite samples, where 100% of the material was recycled in each cycle with same processing parameters.

**Figure 1:**
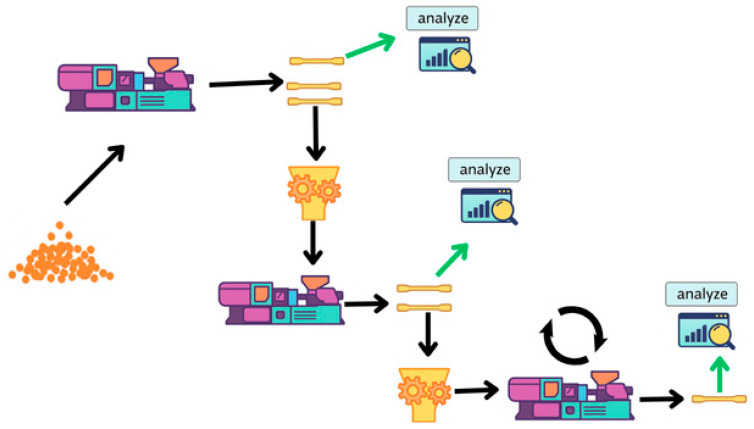
Process of sample preparation during mechanical recycling. Black arrows indicate the processing sequence, and green arrows show the sampling points for analysis.

The injection molding steps applied during the mechanical recycling process of virgin PK composites reinforced with 15 wt% (PK15GF) and 30 wt% (PK30GF) glass fibers are illustrated in detail in Figure 1. As shown, the injection-molded samples were produced in plate form for subsequent mechanical testing. Prior to molding, all granules were dried in an air-circulating oven at 80 ± 10 °C for 4 h, in accordance with the Technical Data Sheet (TDS). Virgin PK15 and PK30 composites were processed using a FANUC injection molding machine (FANUC Corporation, Oshino-mura, Yamanashi, Japan). The molding parameters were as follows: injection pressure of 600 bar, holding pressure of 900 bar, and injection speed of 80 mm·min^−1^. The temperature profile of the screw zones was set to 230 °C, 235 °C, 240 °C, 240 °C, and 235 °C, with a mold temperature of 85 °C. For the production of plate samples used in mechanical testing, an adjusted injection protocol was applied: injection pressure of 400 bar, holding pressure of 650 bar, and injection speed of 50 mm·min^−1^, while keeping all other parameters constant. Using this procedure, injection-molded specimens were successfully produced from 100% recycled aliphatic polyketone composites containing 15 wt% and 30 wt% glass fibers. The resulting products were prepared in the B1 sample format according to the ISO 20753 standard [24].

### 2.2. Methods

Thermogravimetric analysis (TGA) was performed on both virgin and recycled composite materials using a MOM Derivatograph-C apparatus (MOM, Budapest, Hungary). Samples weighing 8 ± 1 mg were placed in open platinum crucibles and heated from 25 °C to 600 °C at a heating rate of 10 °C·min^−1^ under a continuous oxygen flow of 50 mL/min. The aim of this analysis was to evaluate the thermal oxidative degradation behavior and to assess the influence of recycling cycles and glass fiber content on the thermal stability of the composites. Distinct mass loss steps were observed and attributed to the degradation of the polymer matrix and the formation of inorganic residues.

Differential scanning calorimetry (DSC) analyses were performed using a Setaram DSC131 Evo instrument (Setaram Instrumentation, Caluire-et-Cuire, France). Samples of approximately 4–5 mg were sealed in standard aluminum crucibles and heated from 23 °C to 250 °C at a rate of 10 °C/min under a continuous nitrogen flow of 50 mL/min. Melting parameters, including the peak melting temperature (*T_m_*) and enthalpy of fusion (Δ*H_m_*), were determined from the heating scans. The degree of crystallinity (*X_c_*) was calculated using the following equation:

$$X_{c}(\%)=\frac{\Delta H_{m}}{\Delta {H_{m}}^{0}\times (1-\alpha )}\cdot 100$$

where Δ*H_m_* is the measured melting enthalpy of the sample, Δ*H_m_*^0^ is the melting enthalpy of 100% crystalline polyketone (value: 200 J·g^−1^), and α is the weight fraction of glass fibers in the composite [18].

Dynamic Mechanical Analysis (DMA) was conducted using a METRAVIB DMA 25 (METRAVIB, Toulouse, France) instrument to characterize the viscoelastic properties of the composite samples. Measurements were performed in dual cantilever bending mode at a frequency of 1 Hz and a heating rate of 2 °C/min over a temperature range from −50 °C to 150 °C. Rectangular specimens with typical dimensions of approximately 30 × 10 × 3 mm were tested. The storage modulus (E′), loss modulus (E″), and damping factor (tan δ) were recorded as functions of temperature to assess the material’s mechanical behavior and transitions, including the glass transition temperature (T_g_) [25]. All tests were carried out under a nitrogen atmosphere to minimize oxidative degradation during heating.

Tensile test specimens of type B1 were directly injection molded from the composite materials in accordance with the ISO 527-1 BA standard [26]. Tensile tests were performed at room temperature (23 ± 2 °C) and relative humidity of 50 ± 5% using an Instron 5565 Universal Testing Machine (Instron, Norwood, MA, USA) equipped with a 1 kN load cell. The tests were conducted at a crosshead speed of 100 mm/min, following the ASTM D638 standard [27]. For each composite formulation, average tensile properties were calculated from at least five specimens tested under identical conditions [25,28]. For each composite formulation, five specimens were tested under identical conditions, and the tensile properties are reported as mean values with the corresponding standard deviation (SD).

The melt flow index (MFI) is an important parameter reflecting the flowability and viscosity of molten thermoplastic polymers, which directly impacts processing behavior. According to ISO 1133 [29], a 4–5 g polymer sample is heated to a specified temperature, and a piston loaded with a defined weight forces the molten polymer through a standardized die. The mass of polymer extruded over 10 min is measured and expressed in grams per 10 min (g/10 min) [30,31]. Higher MFI values indicate lower viscosity, favoring injection molding, whereas lower MFI values correspond to higher viscosity, suitable for extrusion or blow molding processes. In this study, the melt volume flow rate of polyketone was measured using an INSTRON CEAST MF20 (Instron, Torino, Italy) instrument at 240 °C under a 2.16 kg load, following ISO 1133 specifications. Five measurements were performed per condition, and MFI values are reported as mean ± SD.

Fourier Transform Infrared Spectroscopy (FT-IR) analyses were performed using a Gladi ATR accessory with a diamond crystal on a Bruker Tensor 27 spectrometer (Bruker Corporation, Billerica, MA, USA). Measurements were conducted on virgin PK15GF and PK30GF samples, as well as on samples produced from the original virgin granulated materials. Additionally, mechanically recycled samples subjected to one and five recycling cycles with 100% recycled material content were analyzed. Spectra were recorded in absorbance mode over the wavenumber range of 400–4000 cm^−1^ with a spectral resolution of 4 cm^−1^. Each spectrum represents an average of 128 scans collected at room temperature using a deuterated triglycine sulfate (DTGS) detector [32]. The instrument was equipped with a KBr beamsplitter, suitable for mid-infrared measurements. Data acquisition and processing were performed using OPUS software (version 9.0, Bruker Corporation, Billerica, MA, USA).

## 3. Results and Discussion

### 3.1. Thermogravimetric Analysis of Virgin and Recycled PK-GF Composites

TGA was used to investigate the effect of the recycling cycle and glass fiber content on the thermal stability of virgin PK-GF15 and PK-GF30 composite. Thermal stability of the composites was evaluated through the TGA curves, which show the change in weight of the samples with temperature and the differential thermogravimetry (DTG) curves indicating the weight loss rate, which is a derivative of the TGA chart. Figure 1 shows the TGA and DTG curves of virgin PK-GF15, PK-GF30 and recycled composites, and also the correlative data, the temperature of 5% weight loss (*T*_5_), the temperature of 10% weight loss (*T*_10_), the temperature of 50% weight loss (*T*_50_), the temperature of maximum weight loss (*T*_max_), and the residue percentage at 600 °C are given Table 2. As seen in the TGA curves, all composites displayed a similar one-step thermal degradation process.

**Figure 2:**
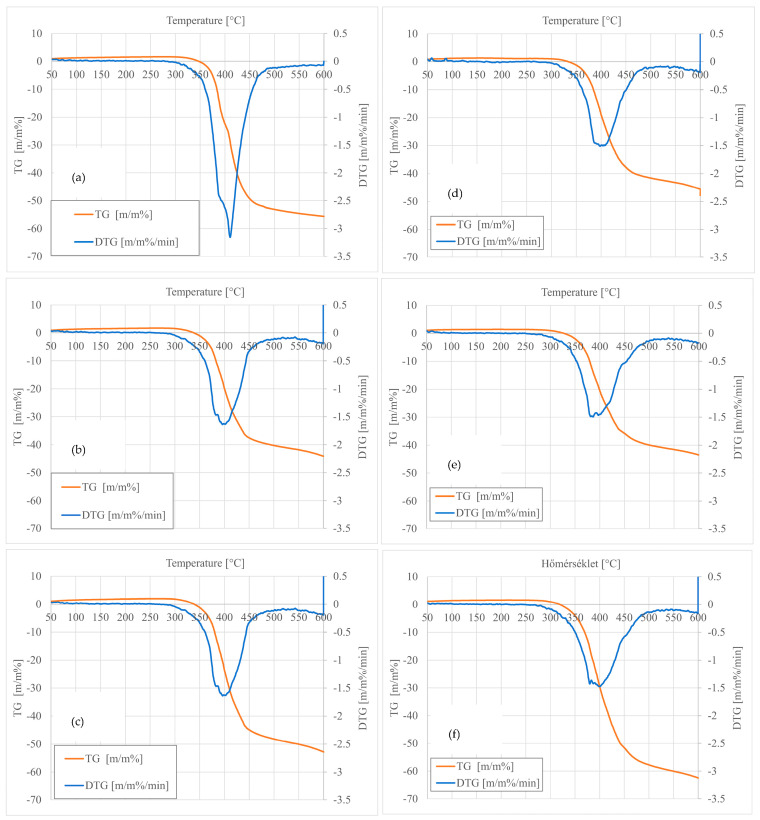
TGA and DTG curves of PK-GF composites. (**a**) Virgin composites (PK15GF, (**b**) Recycled composites (PK15GFREC1), (**c**) Recycled Composite (PK15GFREC5), (**d**) Virgin composites (PK30GF), (**e**) Recycled composite (PK30GFREC1), and (**f**) Recycled composite (PK30GFREC5).

Thermogravimetric analysis (TGA) and derivative thermogravimetry (DTG) were employed to evaluate the thermal decomposition behavior of virgin and recycled PK-GF15 and PK-GF30 composites. As is standard practice in TGA, measurements were performed on representative single samples per composition, as the technique provides consistent and reproducible degradation profiles for homogeneous materials. The TGA curves illustrate the mass loss of the samples as a function of temperature, while the DTG curves represent the rate of this loss. Figure 2 presents the TGA and DTG curves for virgin and recycled composites, and Table 2 summarizes the key decomposition parameters: temperature at 5% (*T*_5_), 10% (*T*_10_), and 50% (*T*_50_) weight loss, temperature at maximum degradation rate (*T_max_*), and the char residue at 600 °C.

The virgin composites (PK15GF and PK30GF) exhibited higher initial decomposition temperatures compared to their recycled counterparts. For example, *PK15GF* had a *T*_5_ of 374.16 °C, which decreased to 369.73 °C in *PK15GFREC5*. Similarly, *PK30GF* showed a *T*_5_ of 372.21 °C, while *PK30GFREC5* dropped to 358.34 °C. These reductions suggest that thermal stability is compromised during recycling, likely due to polymer chain scission and the accumulation of degradation by-products.

DTG curves revealed a shift in *T_max_* values, with virgin samples decomposing at higher temperatures (*PK15GF*: 410.81 °C; *PK30GF*: 405.65 °C) than recycled ones (*PK15GFREC1*: 402.37 °C; *PK30GFREC1*: 382.61 °C). This shift indicates altered degradation kinetics, possibly due to changes in filler–matrix interactions or the presence of lower molecular weight fractions in recycled materials.

Char residue values varied significantly among the samples. Notably, *PK30GFREC5* exhibited the highest residue (62.4%), exceeding the nominal glass fiber content of the composite. This discrepancy may be attributed to the formation of thermally stable carbonaceous structures during repeated recycling cycles. Such residues can result from incomplete polymer decomposition under inert conditions, the presence of sizing agents on the glass fibers, or filler agglomeration, all of which may act as thermal barriers and enhance apparent thermal resistance.

*T*_50_ values were only available for selected samples. Interestingly, *PK15GFREC5* showed a notably high *T*_50_ of 552.14 °C, indicating delayed mass loss and improved thermal endurance. This may be due to the redistribution of glass fibers or the formation of more stable degradation intermediates during multiple recycling cycles.

Overall, virgin samples displayed smoother and more defined decomposition profiles, while recycled composites exhibited broader DTG peaks and earlier onset of mass loss. The broadening of DTG peaks suggests a more heterogeneous degradation process, likely caused by multiple degradation pathways and uneven filler dispersion introduced during recycling.

The TGA and DTG analyses demonstrate that repeated mechanical recycling influences the thermal stability and degradation behavior of PK-GF composites, with notable changes in decomposition temperatures and char residue formation, highlighting the complex interplay between polymer structure, filler content, and processing history. Additionally, the elevated char residue may be partially attributed to the accumulation of thermally stable organic fragments formed during repeated processing. These fragments, resulting from recombination and limited crosslinking, resist complete decomposition and contribute to the residual mass. Such behavior is more pronounced in the PK30GF system, where higher filler content promotes thermal shielding and inhibits full oxidative breakdown.

### 3.2. Differential Scanning Calorimetry (DSC) of Virgin and Recycled PK-GF Composites

**Figure 3:**
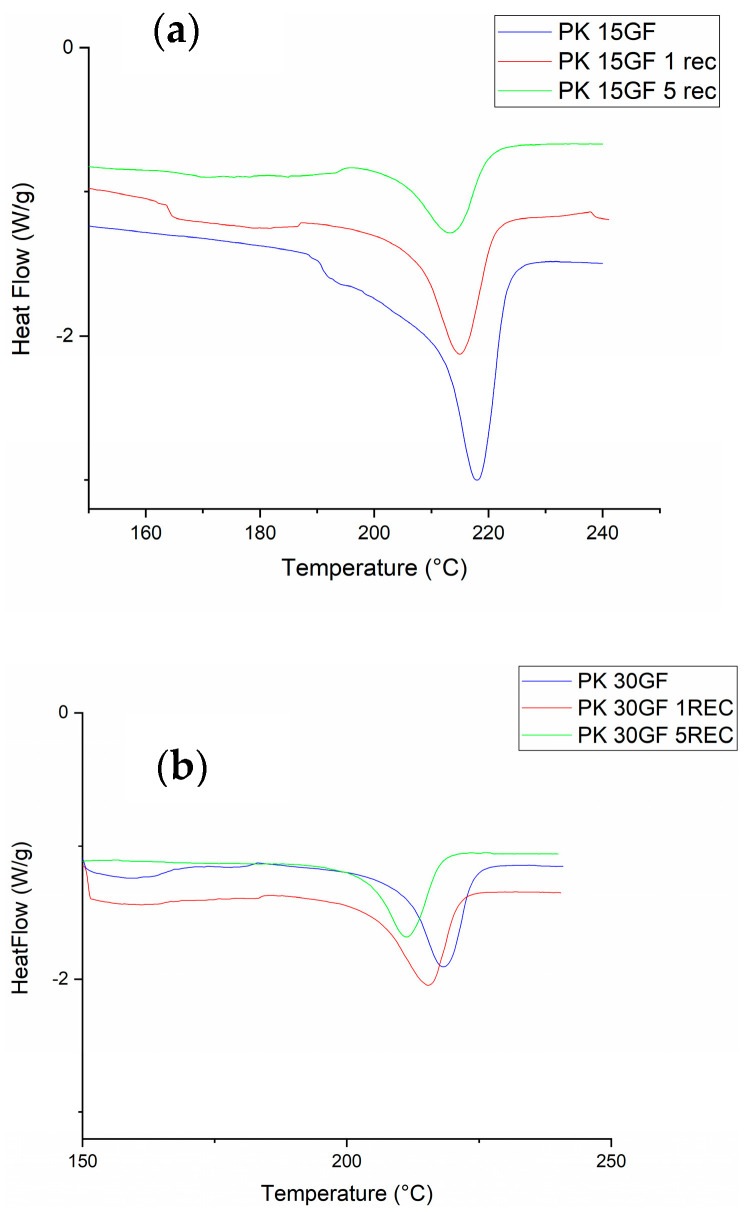
DSC melting thermograms of virgin and recycled PK glass fiber-reinforced composites based on mechanical recycling. (**a**) PK-GF15 series; (**b**) PK-GF30 series.

Differential Scanning Calorimetry (DSC) was performed to investigate the thermal transitions and crystallinity changes in virgin and recycled PK-GF15 and PK-GF30 composites. As shown in Figure 3 and summarized in Table 3, this technique provides insight into the melting behavior, crystallization processes, and possible structural modifications induced by repeated mechanical recycling. The DSC curves presented below illustrate the cooling profiles of the samples, allowing for the evaluation of thermal stability and polymer–filler interactions.

For the virgin PK15GF composite, the melting temperature (Tm) and melting enthalpy (ΔHm) were found to be 217.98 °C and 117.60 J·g^−1^, corresponding to a crystallinity degree (X_c_) of 58.8% (Table 3), calculated using a theoretical melting enthalpy of 200 J·g^−1^ for 100% crystalline aliphatic polyketone. After one and five recycling cycles, Tm values slightly decreased to 214.97 °C and 212.26 °C, while ΔHm significantly decreased to 60.19 J·g^−1^ and 48.36 J·g^−1^, corresponding to crystallinity values of 30.1% and 24.2%, respectively. In the case of the PK30GF composite, which contains a higher glass fiber content, the virgin sample showed a similar melting temperature (218.28 °C) but a substantially lower ΔHm of 45.14 J·g^−1^, resulting in a crystallinity degree of only 22.6%. Upon one and five recycling cycles, T_m_ values dropped to 215.36 °C and 211.35 °C, while ΔHm values declined to 43.29 J·g^−1^ and 31.22 J·g^−1^, with corresponding crystallinity degrees of 21.6% and 15.6%. These results suggest that both recycling and increasing glass fiber content have a notable impact on the crystallization behavior of PK composites. The decreasing Tm and ΔHm values after multiple recycling cycles indicate a gradual reduction in crystalline phase formation. This may be attributed to various structural changes occurring during thermal and mechanical reprocessing. In particular, oxidative degradation and potential crosslinking reactions are likely to occur, leading to the formation of irregular or less mobile polymer chains. Such structural disruptions may hinder the chain folding necessary for well-organized crystalline domains, resulting in lower crystallinity. Additionally, the presence of glass fibers influences the crystallization behavior significantly. The virgin PK30GF composite showed much lower crystallinity than PK15GF, despite having a similar melting temperature. This can be explained by the higher filler content, which limits the mobility of polymer chains and reduces the available space for crystalline structure formation. The filler–matrix interactions and the physical hindrance of fiber-rich regions can inhibit regular crystallite growth. Notably, the crystallinity reduction due to recycling was less drastic in the 30% GF system, which already exhibited limited crystallization potential. Overall, the DSC data clearly show that both repeated reprocessing and increasing glass fiber content reduce the crystallinity of aliphatic polyketone composites, most likely due to combined effects of polymer degradation, restricted chain mobility, and heterogeneous nucleation behavior [33].

### 3.3. Dynamic Mechanical Analysis of Virgin and Recycled PK/GF Composites

**Figure 4:**
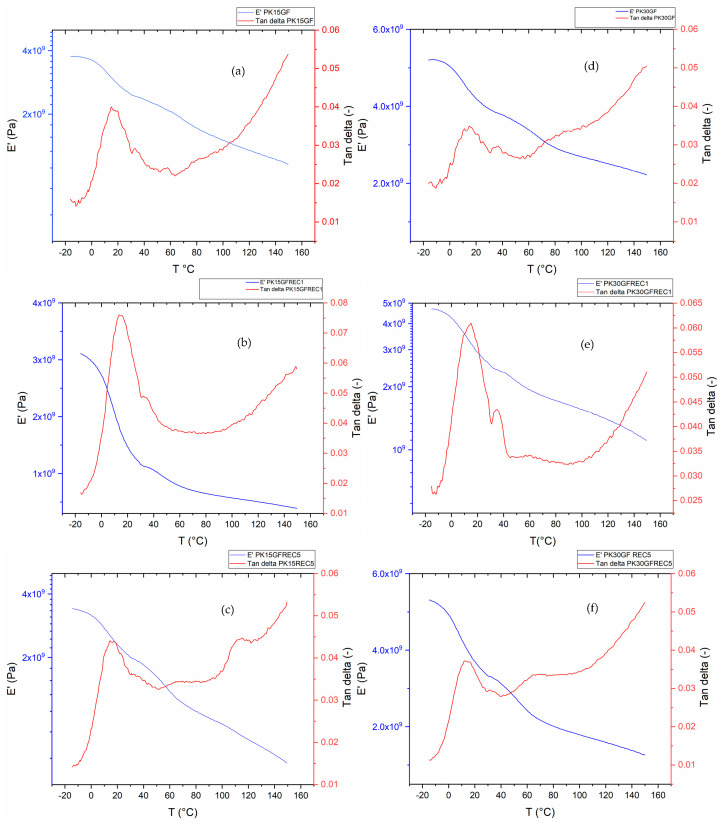
DMA results and T_g_ temperature modification based on the recycling of PK15GF and PK30GF composites. (**a**) PK15GF virgin; (**b**) PK15GF after 1 recycling; (**c**) PK15GF after 5 recyclings; (**d**) PK30GF virgin; (**e**) PK30GF after 1 recycling; (**f**) PK30GF after 5 recyclings.

As shown in Figure 4, dynamic mechanical analysis (DMA) was performed to evaluate the effect of repeated recycling on the glass transition behavior of aliphatic polyketone composites reinforced with 15% and 30% glass fibers. This technique provides insight into the stiffness, damping characteristics, and temperature-dependent mechanical performance of the materials. By analyzing the storage modulus (E′), loss modulus q(E″), and damping factor (tan δ), the effects of repeated mechanical recycling and varying glass fiber content on the dynamic mechanical properties of the composites can be assessed.

Dynamic mechanical analysis (DMA) was performed to evaluate the effect of repeated recycling on the glass transition behavior of aliphatic polyketone composites reinforced with 15% and 30% glass fibers. For the 15 wt% glass fiber-reinforced samples, a modest increase in the glass transition temperature (T_g_) was observed after a single recycling cycle, from 15.10 °C to 16.23 °C. This shift indicates that the molecular mobility within the polymer matrix remains partially constrained, requiring a slightly higher thermal energy input to overcome the restricted segmental motions. The relatively minor change in T_g_ compared to unfilled matrices suggests that glass fiber reinforcement mitigates the effects of polymer chain degradation during processing by providing stress transfer and mechanical stabilization. In contrast, composites containing 30 wt% glass fibers exhibited an almost negligible shift in T_g_ after repeated recycling. The reference, unprocessed sample exhibited a Tg of 15.15 °C, which increased only to 15.20 °C following a single recycling cycle and showed a cumulative increase of 0.45 °C after five cycles. These data demonstrate that higher fiber loadings confer enhanced thermo-mechanical stability to the polymer matrix, maintaining the constrained molecular mobility of the glassy state even after multiple recycling steps. Beyond the Tg shifts, the storage modulus (E′) of both PK15GF and PK30GF composites showed only slight variations across multiple recycling cycles, indicating that the stiffness of the composites was largely preserved. The tan δ peaks remained sharp and of comparable intensity, suggesting that the relaxation processes were not significantly broadened or altered by reprocessing. Taken together, these findings highlight the pivotal role of glass fiber reinforcement in preserving the viscoelastic properties of aliphatic polyketone composites under recycling-induced thermal and mechanical stresses. The results underscore a clear correlation between fiber loading and resistance to processing-induced molecular mobility alterations, providing critical insights for the design of sustainable, mechanically robust polymer composites.

### 3.4. Mechanical Behavior of Recycled PK-15GF and PK-30GF Composites

**Figure 5:**
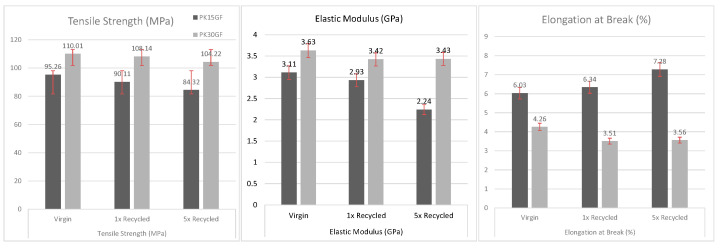
Mechanical behavior of PK 15GF and PK 30GF composite system based on the mechanical recycling.

The mechanical behavior of recycled polymer composites is a critical factor in determining their suitability for structural applications. As shown in Figure 5, the tensile properties of PK-GF15 and PK-GF30 composites are analyzed to assess the impact of repeated mechanical recycling on strength, stiffness, and ductility., the tensile properties of PK-GF15 and PK-GF30 composites are analyzed to assess the impact of repeated mechanical recycling on strength, stiffness, and ductility. These properties provide essential insights into the material’s performance under load and reflect the underlying microstructural changes induced by thermal and mechanical degradation processes.

The mechanical behavior of aliphatic polyketone (PK) composites reinforced with glass fibers was systematically investigated across multiple recycling cycles. Two formulations were studied: PK15GF containing 15 wt% glass fibers, and PK30GF with 30 wt% glass fibers. For the PK15GF system, the virgin composite exhibited a tensile strength of 95 ± 3 MPa, an elastic modulus of 3.1 ± 0.2 GPa, and an elongation at break of 4.0 ± 0.5%. After one recycling cycle, tensile strength and modulus decreased to 90 ± 4 MPa and 2.9 ± 0.3 GPa, respectively, while elongation increased to 6.0 ± 0.8%. Following five recycling cycles, the degradation became more pronounced, with tensile strength and modulus further declining to 84 ± 5 MPa and 2.2 ± 0.4 GPa, respectively, and elongation rising to 7.0 ± 1.0%. These changes are attributed to molecular degradation mechanisms induced by thermal and shear stresses during melt reprocessing. Specifically, chain scission events reduce the average molecular weight, leading to diminished stiffness and strength. Concurrently, the presence of residual oxygen and moisture facilitates radical-mediated recombination, resulting in branching and limited crosslinking. These recombination processes partially restore molecular weight and increase melt viscosity, which manifests macroscopically as enhanced ductility, despite the loss in mechanical rigidity. In contrast, the PK30GF composite demonstrated superior mechanical stability. The virgin material exhibited a tensile strength of 110 ± 2 MPa, an elastic modulus of 3.6 ± 0.1 GPa, and an elongation at break of 6.0 ± 0.7%. After one recycling cycle, only minor reductions were observed: 108 ± 3 MPa in tensile strength and 3.4 ± 0.2 GPa in modulus, with elongation slightly increasing to 6.0 ± 0.9%. Even after five recycling cycles, the composite retained high mechanical integrity, with 104 ± 4 MPa tensile strength, 3.0 ± 0.3 GPa modulus, and 7.0 ± 1.1% elongation at break. The enhanced recyclability of PK30GF is attributed to the dual role of glass fibers. Beyond mechanical reinforcement, the higher fiber content acts as a thermal buffer, mitigating the extent of chain scission by reducing the polymer matrix’s exposure to thermal and shear stresses. Additionally, the fibers serve as physical barriers, limiting polymer chain mobility and inhibiting radical propagation, thereby reducing both degradation and uncontrolled crosslinking. This results in a more resilient molecular architecture and improved load transfer efficiency, which collectively preserve mechanical properties over repeated processing cycles. The mechanical evolution of PK/glass fiber composites during recycling is governed by the interplay between chain scission and recombination mechanisms. While degradation reduces stiffness and strength, recombination and limited crosslinking enhance ductility and melt viscosity. The presence of higher glass fiber content not only reinforces the composite but also stabilizes the polymer matrix, significantly improving its recyclability and long-term mechanical performance.

### 3.5. Melt Flow Property of Recycled PK-15GF and PK-30GF

**Figure 6:**
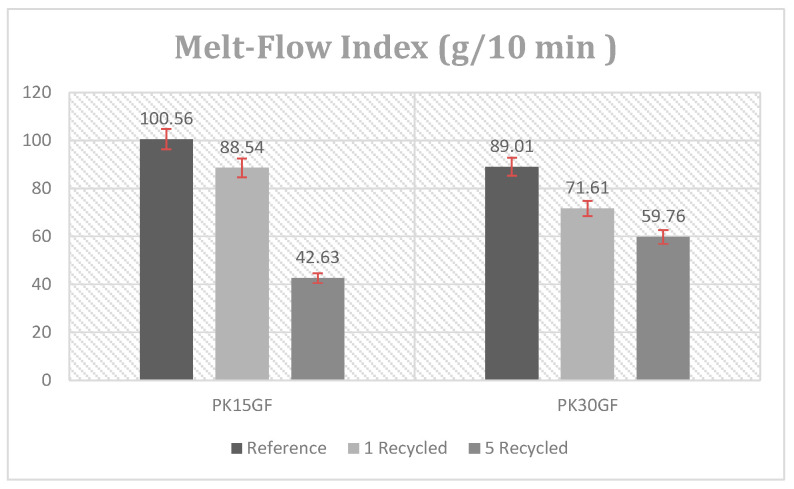
MFI test results of the virgin and recycled PK-GF15 and PK-GF30 composites.

The influence of incorporating the recycled material obtained in 2 different recycling cycles on the melt flow property of PK-15GF and PK-30GF composite was studied by MFI analysis, which is a measure of melt fluidity or viscosity compered the reference non recycled samples. The MFI values obtained as a function of the recycling cycle and recycled material ratio incorporated into the virgin composite are given in Figure 6. As can be seen, the obtained MFI values clearly revealed that the melt flow behavior of the PK composites were significantly affected by the applied mechanical recycling process, the recycled material content in the composite, and the number of recycling cycles.

The melt flow index (MFI) measurements reveal a progressive decline in flowability as a function of recycling cycles, indicating substantial changes in the rheological behavior of the composites. For the PK15GF system, the MFI decreased from 100.56 g/10 min (virgin reference) to 88.54 g/10 min after a single recycling cycle (PK15GFREC1), and further to 42.63 g/10 min following five cycles (PK15GFREC5), corresponding to a 57.6% reduction overall. In contrast, the PK30GF composite exhibited a less pronounced decline—from 89.00 g/10 min (reference) to 71.61 g/10 min (PK30GFREC1), and 59.76 g/10 min after five cycles (PK30GFREC5)—reflecting a 32.9% total decrease. These reductions in MFI correspond to increased melt viscosity, primarily driven by thermomechanical degradation during reprocessing. The dominant mechanism is chain scission, where polymer chains are broken under the influence of thermal and shear stress, leading to a reduction in molecular weight. However, in the presence of residual oxygen or moisture, radical-mediated pathways may become significant, facilitating recombination of chain fragments or even limited branching and crosslinking [34]. Such recombination processes can contribute to increased effective molecular weight, further elevating melt viscosity and intensifying the decline in flowability. The extent and impact of these degradation and recombination processes are strongly influenced by the glass fiber content. In the PK30GF system, the higher filler concentration not only reinforces the matrix mechanically, but also provides thermal buffering and acts as a barrier that limits polymer chain mobility and inhibits radical propagation. This dual role of the glass fibers stabilizes the material against degradation and mitigates the reduction in flow properties during repeated melt processing. The comparatively modest drop in MFI for the PK30GF composite suggests that increasing fiber content by 15 wt% yields a disproportionately greater improvement in resistance to degradation-induced viscosity changes, underscoring the critical role of fiber reinforcement in enhancing the recyclability and processing stability of aliphatic polyketone composites. In contrast to the observed decrease in melt flow index (MFI) and crystallinity in recycled aliphatic polyketone composites, studies on polyamide 6.6 (PA66) have reported an increase in MFI after multiple recycling cycles. For instance observed that the MFI of PA66 composites increased after five recycling cycles, indicating a reduction in molecular weight and an increase in viscosity due to chain scission [34]. This difference in behavior between PA66 and polyketone composites suggests that while PA66 undergoes chain scission upon recycling, leading to increased MFI, polyketone composites may experience crosslinking or chain extension, resulting in decreased MFI and increased viscosity. These contrasting mechanisms highlight the importance of understanding the specific degradation pathways of different polymers during recycling processes [35].

### 3.6. FT-IR Characterization of PK-15GF and PK-30GF

**Figure 7:**
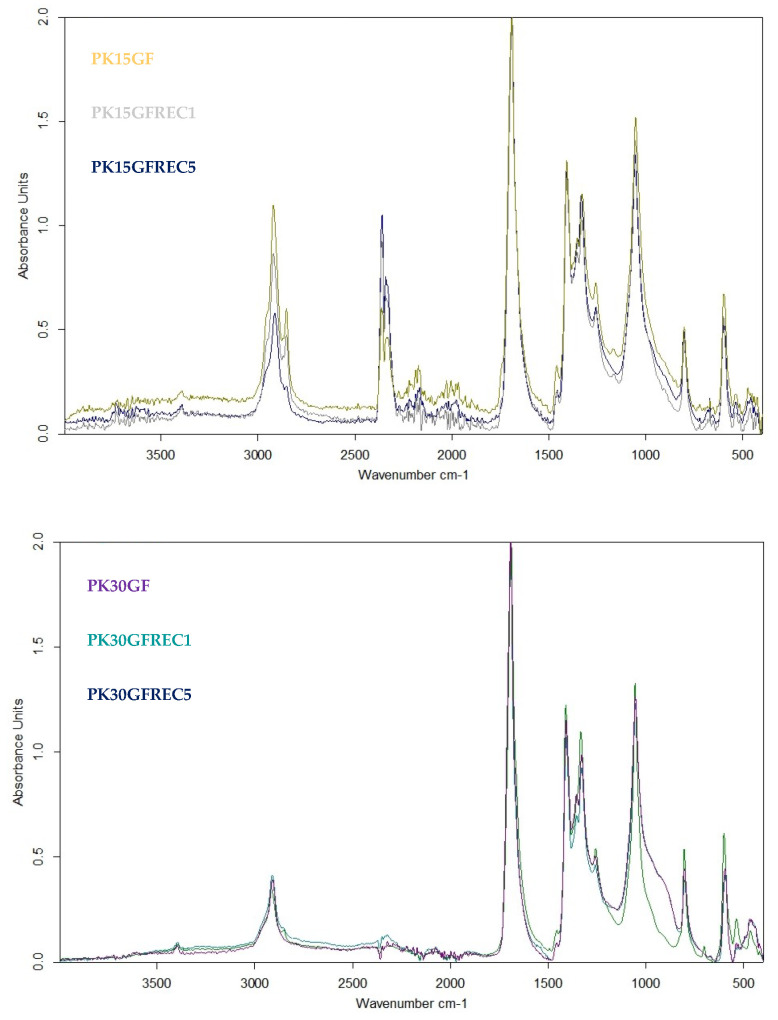
Chemical structure modification based on the FT-IR spectra of PK15GF and PK30GF during the mechanical recycling.

The FT-IR spectra of PK-15GF in the virgin state and after one and five recycling cycles are shown in Figure 7. All major absorption bands characteristic of aliphatic polyketones were consistently present. The spectra exhibited a strong carbonyl stretching vibration at ~1690 cm^−1^, which is the dominant feature of the polyketone backbone. The C–H stretching vibrations of methylene groups appeared between 2850 and 2950 cm^−1^, while the corresponding bending and scissoring vibrations were visible in the 1450–1370 cm^−1^ region. Additional peaks between 1200 and 1000 cm^−1^ were assigned to C–O and C–C skeletal vibrations, reflecting the integrity of the polymer chain [31,32]. Vibrations below 900 cm^−1^ correspond to out-of-plane bending modes, further confirming the structural fingerprint of the material. When comparing the virgin and reprocessed samples, no new absorption bands were detected, indicating the absence of significant chemical byproducts or extensive crosslinking. However, subtle variations were observed. The carbonyl band around 1690 cm^−1^ exhibited slight broadening and reduced intensity after multiple recycling cycles, suggesting minor oxidative modifications and local chain scission. Similarly, changes in the 1200–1000 cm^−1^ region pointed to limited alterations in the C–O stretching environment, consistent with increasing structural heterogeneity. In contrast, the C–H stretching region (2850–2950 cm^−1^) remained essentially unchanged, confirming that the aliphatic backbone was preserved [32]. Overall, the FT-IR results demonstrate that the primary molecular structure of the polyketone matrix remains intact after repeated reprocessing, while minor modifications—primarily oxidation and chain cleavage—contribute to spectral broadening and intensity changes. These observations are consistent with the mechanical and thermal results, highlighting the recyclability and structural robustness of glass fiber-reinforced polyketones [36].

The FT-IR spectra of PK-30GF composites in virgin, once-recycled, and five-times recycled states are presented in Figure 7. The characteristic absorption bands of aliphatic polyketones were consistently detected across all samples. The dominant carbonyl stretching vibration was observed around ~1690 cm^−1^, while methylene C–H stretching bands appeared between 2850 and 2950 cm^−1^, accompanied by bending vibrations in the 1450–1370 cm^−1^ region. Additional absorptions in the 1200–1000 cm^−1^ range correspond to C–O and skeletal vibrations, and several peaks below 900 cm^−1^ were assigned to out-of-plane deformations. These features confirm that the primary chemical structure of the polymer backbone was preserved after repeated recycling. Compared with the virgin material, the recycled samples exhibited only minor changes in the spectra. Subtle variations in the carbonyl band (~1690 cm^−1^) were observed, including slight broadening and intensity changes, which may be attributed to limited oxidative modification and chain scission. Similarly, the C–O stretching region showed modest intensity differences with increasing recycling cycles, suggesting the development of local heterogeneity within the matrix. Importantly, no new absorption bands emerged in the spectra, confirming the absence of significant chemical byproducts or extensive crosslinking reactions. Overall, the FT-IR analysis of PK-30GF demonstrates that the backbone of the polymer matrix remains stable after multiple mechanical recycling cycles. The minor spectral modifications detected indicate that, while slight structural rearrangements occur, the presence of higher glass fiber content contributes to maintaining structural integrity and mitigating degradation compared to lower filler levels. These results highlight the positive influence of glass fiber reinforcement on the chemical stability and recyclability of aliphatic polyketone composites.

## 4. Conclusions

This study provides a comprehensive evaluation of the mechanical recycling behavior of glass fiber-reinforced aliphatic polyketones, specifically ethylene–propylene copolymer-based matrices containing 15 and 30 wt% glass fibers. By systematically combining thermal, mechanical, rheological, and spectroscopic analyses across virgin, single-cycle, and five-cycle recycled states, the study elucidates the interplay between fiber content, degradation pathways, and performance retention during repeated melt reprocessing.

The higher glass fiber content significantly enhances recyclability and performance retention. PK30GF composites retained tensile strength, modulus, and viscoelastic stability to a much greater extent than PK15GF counterparts, reflecting the dual role of glass fibers as mechanical reinforcements and stabilizing agents against thermal and oxidative degradation. Recombination and limited crosslinking processes play a critical role in the mechanical evolution of recycled composites. While chain scission reduces stiffness and strength, radical-mediated recombination partially restores molecular weight and increases melt viscosity, contributing to enhanced ductility and structural integrity. A strong correlation was observed between melt viscosity changes and mechanical property retention. MFI results revealed that increased viscosity upon repeated processing is less pronounced at higher fiber fractions, underscoring the importance of filler content in dictating rheological stability and long-term usability.

Thermal analyses (TGA/DSC) revealed gradual shifts in decomposition and crystallization behavior, whereas DMA and tensile testing highlighted the capacity of glass fibers to preserve stiffness and load-bearing ability despite multiple recycling cycles. Complementary FT-IR spectroscopy confirmed the chemical robustness of the PK backbone, with only subtle oxidation-related modifications observed after extended recycling.

Taken together, these findings establish glass fiber-reinforced aliphatic polyketones as promising candidates for sustainable engineering applications requiring durability under circular economy frameworks. The superior performance retention of PK30GF composites suggests that strategic adjustment of fiber content can effectively mitigate recycling-induced deterioration and extend material lifetimes. From a broader perspective, this study not only advances the understanding of polyketone recyclability but also provides design guidelines for tailoring high-performance thermoplastic composites toward long-term reusability in structural and automotive sectors.

## Figures and Tables

**Table 1:** Compositions of reference and recycled PK GF15 and-GF30 composite samples.

Sample Code	Material Content and Description
PK15GF	Original virgin granules of polyketone with 15% glass fiber content
PK15REC1	100% 1 cycled recycled polyketone with 15% glass fiber content
PK15REC5	100% 5 cycled recycled polyketone with 15% glass fiber content
PK30GF	Original virgin granules of polyketone with 30% glass fiber content
PK30REC1	100% 1 cycled recycled polyketone with 30% glass fiber content
PK30REC5	100% 5 cycled recycled polyketone with 30% glass fiber content

**Table 2:** Thermal decomposition data of virgin and recycled PK-GF15 and PK-GF30 composites.

Sample	T_5_^a^ (°C)	T_10_ ^b^ (°C)	T_50_ ^c^ (°C)	T_max_ (°C)	^d^ Char Residue
PK15GF	374.16	382.25	456.22	410.81	55.62
PK15GFREC1	372.33	382.72	-	402.37	44.15
PK15GFREC5	369.73	379.24	552.13	397.96	52.83
PK30GF	372.21	384.75	-	405.65	45.61
PK30GFREC1	365.82	379.81	-	382.61	43.44
PK30GFREC5	358.34	371.50	444.21	397.63	62.42

^a, b, c^ Temperatures corresponding to 5%, 10%, and 50% weight loss, respectively. - indicates that 50% mass loss was not achieved within the measured temperature range. ^d^ Char residue obtained at 600 °C, expressed as a percentage of the initial sample mass. Values exceeding the nominal glass fiber content may result from the formation of thermally stable degradation products during recycling.

**Table 3:** DSC data of virgin and recycled PK composites with glass fiber.

Sample	*T*_m_ (°C)	Δ*H*_m_ (J·g^−1^)	*X*_c_ (%)
PK15GF	217.98	117.60	58.80
PK15GF1REC	214.97	60.18	30.09
PK15GF5REC	212.26	48.35	24.18
PK30GF	218.28	45.14	22.57
PK30GF1REC	215.36	43.28	21.64
PK30GF5REC	211.35	31.21	15.61

## Data Availability

The original contributions presented in this study are included in the article. Further inquiries can be directed to the corresponding author.
